# The relationship between personality traits and health-related quality of life after mild-to-moderate traumatic brain injury

**DOI:** 10.1186/s12883-025-04153-0

**Published:** 2025-04-11

**Authors:** Benedikte Å. Madsen, Silje C. R. Fure, Cecilie Røe, Daniel Løke, Marianne Løvstad, Nada Andelic, Emilie Isager Howe

**Affiliations:** 1 Department of Physical Medicine and Rehabilitation, Oslo University Hospital, Oslo, Norway; 2 Institute of Clinical Medicine, Faculty of Medicine, University of Oslo, Oslo, Norway; 3 Research Center for Habilitation and Rehabilitation Models and Services (CHARM), Institute of Health and Society, University of Oslo, Oslo, Norway; 4 Department of Research, Sunnaas Rehabilitation Hospital Trust, Nesoddtangen, Norway; 5 Department of Psychology, Faculty of Social Sciences, University of Oslo, Oslo, Norway

**Keywords:** Traumatic brain injury, Health-related quality of life, Post-concussion symptoms, Neuroticism, Extraversion, Conscientiousness, Rehabilitation after concussion

## Abstract

**Background:**

Traumatic brain injury (TBI) causes major societal burden and may negatively influence an individual’s health-related quality of life (HRQoL). Personality factors have been linked to persistent post-concussion symptoms (PPCS), and PPCS have been found to affect HRQoL. However, there is a knowledge gap concerning the association between personality traits and HRQoL after mild-to-moderate TBI. Thus, this study aims to investigate the association between personality traits and HRQoL in patients with mild-to-moderate TBI at 15 months post-injury, while controlling for socio-demographic characteristics, injury-related factors and symptom burden.

**Methods:**

Data from 86 participants with mild- to-moderate TBI from a previous randomised controlled trial were analysed. Sociodemographic, injury-related and psychological factors were recorded 2–3 months post-injury. Personality traits were measured at 15 months post-injury with The NEO Five-factor Inventory-3. The Quality of Life after Brain Injury– Overall Scale (QOLIBRI-OS) and the EuroQol-visual analogue scale (EQ-VAS) were used to measure HRQoL at 15 months post-injury. Two separate multiple linear regression models were performed for the outcome variables; QOLIBRI-OS (model 1) and EQ-VAS (model 2).

**Results:**

The factors associated with lower HRQoL were more severe PPCS, higher levels of the personality traits neuroticism and conscientiousness (model 1), female sex and being single/living alone (model 2). Higher levels of the personality trait extraversion were associated with higher HRQoL in both models.

**Conclusion:**

The results highlight how non-injury factors may be associated with recovery and HRQoL after TBI. Considering personality factors may be helpful when identifying individual risk and protective factors for outcomes after mild-to-moderate TBI.

**Supplementary Information:**

The online version contains supplementary material available at 10.1186/s12883-025-04153-0.

## Background

Traumatic brain injury (TBI) is associated with long-term limitations in functioning, activity of daily life and participation, reduced health-related quality of life (HRQoL) and profound socioeconomic consequences [1, 2]. Approximately 30% of individuals with mild or moderate TBI experience symptoms that persist more than three months after injury, referred to as persistent post-concussion symptoms (PPCS) [1, 3, 4], with the extensive majority (up to 90%) being mild TBI (mTBI). Pre-injury factors, such as anxiety, depression, previous mental health problems and headache [3, 5–7] are of importance for the risk of PPCS and prolonged recovery after mTBI [8, 9]. More nuanced approaches are lacking to better identify individuals at risk of prolonged symptoms and facilitate early, targeted treatment. Hence, it is important to gain further insight into non-injury factors predicting mTBI recovery, such as psychological characteristics [10].

Problems with persisting symptoms and resuming pre-injury occupational status can lead to reduced social integration and quality of life (QoL)^1^ after mTBI [2, 12]. Research on functional outcomes after TBI often use instruments that fail to include the patient´s subjective experience [13]. Increased awareness of the importance of measuring the subjective burden of the injury, has led to a focus-shift toward HRQoL in TBI research [14]. HRQoL reflects a patient´s perception of how a disease and its treatment affect the physical, psychological, and social aspects of his or her daily life [15]. HRQoL can be measured with a generic or disease-specific instrument [13]. The EuroQol visual analogue scale (EQ VAS) is a generic instrument applicable across a wide range of conditions, and is commonly used within the field of TBI [16, 17]. The Quality of Life after Traumatic Brain Injury Overall scale (QOLIBRI-OS) [18] is a disease-specific measure of HRQoL, which is more sensitive to a patient´s specific health condition and the consequences of it. Although disease-specific instruments may not allow for comparison with healthy individuals, reference values in the general population have recently been developed for the QOLIBRI-OS [19]. Research has shown that a third of patients with TBI (including mild and moderate), reported reduced HRQoL up to 10 years after injury [20], and that patients with mTBI and PPCS have lower HRQoL compared to patients without PPCS [2]. Still, we lack a comprehensive understanding of the relationship between PPCS, their psychological risk factors and HRQoL after mTBI.

Recovery after mTBI seems to be affected by both mental health and personality factors [21–24]. The American Psychological Association (APA) defines personality traits as; relatively stable, consistent and enduring internal characteristics that are inferred from a pattern of behaviours, attitudes, feelings, and habits in an individual [25]. According to the five-factor model (FFM) of personality, there are five broad domains of personality; neuroticism, extraversion, openness to experience, agreeableness and conscientiousness [26]. The FFM is validated across cultures [27, 28] and holds predictive value for health outcomes [29]. TBI research has so far mainly focused on the link between higher levels of neuroticism (i.e. tendencies toward negative affect including sadness, anxiety, and anger [30]) and more severe PPCS [22, 23, 31, 32], anxiety and depression symptoms. To our knowledge, no studies have explored the association between personality traits and HRQoL after mTBI. However, studies of other patient populations have shown associations between personality and HRQoL. A systematic review [33] including individuals with various health states, found a consistent relationship between personality and HRQoL, with personality characteristics often outweighing the effects of demographic, social and clinical factors. The review showed that higher levels of extraversion, agreeableness, openness and conscientiousness were related to better HRQoL, while greater neuroticism and negative affectivity were associated with poorer HRQoL [33]. Lemos and collegues [34] showed the importance of personality and emotional symptoms on HRQoL in patients with unruptured intracranial aneurysms, and stressed the importance of developing strategies for psychological counseling for these patients. More insight is needed to understand the relationship between personality traits and HRQoL in patients with TBI. This could aid in identifying risk and protective factors for patients, and development of targeted treatment strategies after mild-to-moderate TBI. Thus, the aim of this study was to investigate the association between personality traits and HRQoL while controlling for socio-demographic characteristics, injury-related factors and symptom burden in patients with mild-to-moderate TBI at 15 months post-injury. We chose to include both a generic and disease specific measure of HRQoL as the two types of instruments measures somewhat different aspects of HRQoL. The generic instrument can be applied to both healthy and non-healthy individuals, while the disease specific measure is specifically designed to capture the impact of TBI on HRQoL. Based on exisiting literature, we hypothesized that higher levels of neuroticism would be associated with lower HRQoL.

## Methods

### Study design

The data in the current study was collected in a previous randomized controlled trial (RCT) carried out between 2017 and 2020 [35]. The RCT was registered at at the US National Institutes of Health (ClinicalTrials.gov, #NCT03092713), and compared the effectiveness of a combined cognitive and vocational intervention to multidisciplinary treatment as usual on return to work (RTW) after mild-to-moderate TBI. For a total of 6 months, the participants received either the study intervention or treatment as usual. Results from the RCT have been published previously [36, 37]. There were no statistically significant differences between the two groups regarding HRQoL at 15 months post-injury and the participants are therefore analysed as one cohort in the current study. The RCT was approved by the Regional Committee for Medical and Health Research Ethics in South-Eastern Norway (#2016/2038), and a notification of amendment for secondary personality trait analyses was approved on 28 February 2022 (6481).

### Participants

In the original RCT, 116 participants were included from a specialised TBI rehabilitation outpatient clinic at the Department of Physical Medicine and Rehabilitation at Oslo University Hospital (OUH), Norway. Eligible patients referred from the emergency room, neurosurgical department or by general practitioners were assessed according to the following inclusion criteria: mild-to-moderate TBI 2–3 months previously as assessed by a Glasgow Coma Scale score of 10–15, loss of consciousness for < 24 h, and posttraumatic amnesia for < 7 days, 18–60 years of age, residing in Oslo or Akershus county, employed at least 50% at the time of injury and sick listed 50% or more at the time of inclusion due to PPCS, assessed with the Rivermead Post-Concussion Symptoms Questionnaire (RPQ) [38]. Severe psychiatric or neurological illness, active substance abuse and insufficient Norwegian language skills were exclusion criteria. mTBI was defined by the criteria developed by the American Congress of Rehabilitation Medicine [39] and assessed by reviewing medical records or established at the time of screening for study eligibility. In the current study, we analysed data from participants with available responses on the NEO Five-Factor Inventory-3 (NEO-FFI-3) [40, 41] measure of personality (*N* = 87). In a previous study [42], we established that the baseline characteristics of these 87 patients did not differ significantly from the patients who had not responded to the personality inventory, except for years of education. Additionally, one participant had missing response on the EQ VAS measure of health, leaving a total of 86 out of the original 116 participants to be included in the current study.

### Procedures

All participants provided written informed consent. Sociodemographic, injury- and work-related information were collected during an interview [35]. Clinical characteristics were self-reported or obtained by reviewing medical records. Data collection was performed at a baseline assessment (2–3 months post-injury) and at 12 months follow-up after study inclusion (i.e. approximately 15-months post-injury). A clinical psychologist or a medical doctor performed the baseline assessment at the outpatient clinic at the Department of Physical Medicine and Rehabilitation, OUH. Follow-up assessments were conducted by blinded outcome assessors.

### Outcome measures

The main outcome measure in this study was HRQoL, assessed with two instruments: the generic EuroQol visual analogue scale (EQ VAS) [16, 17] and the disease-specific Quality of Life after Brain Injury– Overall Scale (QOLIBRI-OS) [18]. The participants completed both questionnaires at 15 months post-injury. QOLIBRI-OS consists of six items (i.e. physical, emotional, cognition, social and daily life, and overall satisfaction with the situation and future perspective), rated from 1 (“Not at all”) to 5 (“Very”). The sum of the six items was converted arithmetically to a percentage scale, with 0 representing the lowest possible HRQoL and 100 the best possible HRQoL [18]. QOLIBRI-OS scores below 52 represent low or impaired HRQoL [43]. The EQ VAS records the patient’s self-rated health on the day in question on a visual analogue scale ranging from 0 (“The worst health you can imagine”) to 100 (“The best health you can imagine”) [16]. A recent study provided Norwegian population norms for the EQ VAS, with mean scores of 77.9 (SD 18.3) [44].

### Independent variables

Sociodemographic variables were age (years), sex (male/female), education (years), marital status (married or cohabitating vs. being single or living alone) and the presence of children in the household (yes/no).

Injury-related variables included cause of injury, injury severity (mild/moderate TBI), the presence of traumatic intracranial findings on computed tomography (CT) or magnetic resonance imaging (MRI) (yes/no) and extracranial injury (yes/no).

PPCS were measured with the RPQ [38]. Total score at baseline was included to describe symptom burden. The RPQ asks the patients to score 16 post-concussive symptoms, compared to before the injury, on a 5-point Likert scale ranging from 0 (“Not experienced”) to 4 (“Severe problem”), adding up to a total score of 0–64. As recommended, scores of 1 (“No more of a problem than before”) were not included in the total score [38]. The RPQ correlated highly with symptom measures of depression (Patient Health Qestionnaire-9) [45], anxiety (Generalized Anxiety Disorder-7) [46] and post-traumatic stress (Post-Traumatic Symptom Scale-10) [47] in this sample (Pearson’s *r* = 0.6–0.7, *p* < 0.002), and these measures were therefore not included in the analyses.

Personality traits were measured with the NEO-FFI-3 [40, 41], assessed at 15 months post-injury. The NEO-FFI-3 is a short version of the NEO Personality Inventory-3 (NEO-PI-3) [48]. It comprises 60 items and measures five dimensions of personality: neuroticism (e.g., the tendency to experience negative affect versus emotional stability); extraversion (e.g., sociability, assertiveness, excitement-seeking, and optimism versus reserved, independent, and even-paced); openness to experience (e.g., active imagination, attentiveness to inner feelings, and intellectual curiosity versus conventional and conservative); agreeableness (e.g., trustful, altruistic, and modest versus skeptical and antagonistic); and conscientiousness (e.g., purposeful, determined, and organized versus unreliable and spontaneous). Each dimension is assessed by 12 statements, rated on a 5-point Likert scale ranging from 0 (strongly disagree) to 4 (strongly agree). The raw scores were converted to T-scores (mean of 50, standard deviation = 10) using sex-corrected norms from a Norwegian population-based sample [40]. T-scores of 56 or higher are considered high, 45 to 55 are considered average, and 44 or below are considered low. The NEO-FFI-3 has previously been validated in the Scandinavian context [49, 50].

### Statistical analysis

Statistical analyses were performed in Stata version 16 [51]. Baseline characteristics of patients and variables included in the regression analyses are presented with descriptive statistics. Multivariable linear regression was used to develop two separate models for the outcome variables QOLIBRI-OS (model 1) and EQ VAS (model 2), both measured at 15 months post-injury. First, a global model for each of the outcomes was established with the independent variables (age, sex, education, marital status, children in household, all five personality traits, injury severity, traumatic intracranial findings on CT/MRI, extra-cranial injuries and PPCS at baseline), chosen from literature and expert opinion, as recommended [52]. The two global models were then reduced to the best-fitting models using manual backward elimination and Akaike information criterion corrected (AICc) to assess goodness of fit in each step. An increase in AICc served as stopping point for the backward elimination. Additionally, we controlled for the variable representing intervention vs. control group in each regression model. This was done to control for possible effects of the treatment in the original RCT, but showed no significance and was therefore not included in the final models. Normality of the residuals was controlled using a Q–Q plot (see Supplementary Materials 1), and multicollinearity was checked by the variable inflation factor. R^2^ and adjusted R^2^ represents the amount of variance in HRQoL at 15 months post-injury, explained by both model 1 and 2. To check for internal validity, the models were run with 1,000 bootstrap repetitions. Statistical significance was set to *P* < 0.05 for all analyses.

## Results

### Sample characteristics

The participant’s mean age was 43 years (SD = 10) and 59% were female. Mean years of education was 16 (SD = 2) and 94% had sustained an mTBI. The most common cause of injury was falls (43.5%). At baseline, the mean RPQ score was 28 (SD = 11), indicating an overall high symptom burden. The mean QOLIBRI-OS score was 47 (SD = 22) (indicating impaired HRQoL) and the mean EQ VAS score was 53 (SD = 17) (considerably below the mean EQ VAS score of the general population). Mean scores of all five personality traits were within the average of the general population. See Table 1 for further details on sample characteristics. The characteristics of participants with mild and moderate TBI, respectively, are presented in Supplementary Materials 1.

**Table 1:** Characteristics of the participants (*N* = 86)

		Mean (SD)	*N* (%)
Sociodemographic factors	Age	43 (10)	
	Female		51 (59)
	Years of education	16 (2)	
	Married/cohabitant		55 (66)
	Children in the household		46 (53)
Cause of injury	Fall		37 (43.5)
	Exposure to inanimate mechanical forces		17 (20)
	Transport		15 (17)
	Sports		12 (14)
	Violence		4 (4.5)
	Unknown		1 (1)
Injury severity	Mild TBI		81 (94)
	Moderate TBI		5 (6)
LOC	Yes		31 (36)
PTA	Yes		41 (48)
Intracranial findings on CT/ MRI	Yes		20 (23)
Extracranial injury	Yes		41 (48)
Symptom burden (at baseline)	RPQ	28 (11)	
HRQoL (at 15 months post-injury)	QOLIBRI-OS	47 (22)	
	EQ VAS	53 (17)	
Personality traits (at 15 months post-injury)	Neuroticism	49.1 (10.4)	
	Extraversion	44.4 (10.7)	
	Openness to experience	47.5 (9)	
	Agreeableness	54.1 (9.4)	
	Conscientiousness	55.8 (7.6)	

Abbreviations: CT, computed tomography; MRI, magnetic resonance imaging; LOC, loss of consciousness; PTA, posttraumatic amnesia; RPQ, Rivermead Post-Concussion Symptoms Questionnaire; HRQoL, Health-related quality of life; QOLIBRI-OS, The Quality of Life after Traumatic Brain Injury Overall scale; EQ VAS, EuroQol Visual Analogue Scale

### Association between personality traits and health related quality of life

The variables significantly associated with lower HRQoL at 15 months post-injury as measured by QOLIBRI-OS (model 1), were more severe PPCS at baseline and higher levels of neuroticism, and conscientiousness, while higher levels of extraversion on the other hand, were significantly associated with higher scores on the QOLIBRI-OS (Table 2). With a regression coefficient of -0.83, conscientiousness was the personality trait with the strongest association with QOLIBRI-OS, followed by neuroticism (-0.57) and extraversion (0.45). Model 1 explained 30% of the variance in QOLIBRI-OS at 15 months post-injury. Bootstrap analysis confirmed all the significant associations, and all values are presented after bootstrapping in Table 2. Openness to experience and agreeableness showed no significant association with QOLIBRI-OS. The former was thus omitted from the final model to improve model fit. All the variable inflation factor values were between 1.04 and 1.98, indicating no issues of multicollinearity.

**Table 2:** Model 1: the association between demographics, PPCS, personality traits and QOLIBRI-OS (*N* = 86)

Variable	Unstd. Coefficient	Bootstrap Std. err.	*p*- Value	Normal-based C.I. 95%
Female	-7.09	4.7	0.132	-16.3 to 2.13
Children in household^1^	-5.41	3.94	0.170	-13.13 to 2.32
Baseline RPQ score	-0.53	0.21	**0.010**	-0.94 to -0.13
Neuroticism	-0.57	0.26	**0.032**	-1.01 to -0.05
Extraversion	0.45	0.21	**0.033**	0.04 to 0.89
Agreeableness	0.36	0.21	0.090	-0.06 to 0.78
Conscientiousness	-0.83	0.34	**0.013**	-1.49 to -0.17
Constant	119.11	27.95	0.000	64.33 to 173.9
R^2^		0.35		
Adj. R^2^		0.30		

^1^Children in household: 0– No; 1– Yes. Abbreviations: PPCS, Persistent post-concussion symptoms; QOLIBRI-OS, The Quality of Life after Traumatic Brain Injury Overall scale; RPQ, Rivermead Post-Concussion Symptoms Questionnaire. Bold indicates statistical significance

The variables significantly associated with lower HRQoL at 15 months post-injury, measured by EQ VAS (model 2), were female sex and being single/living alone, whereas higher levels of extraversion were significantly associated with higher scores on the EQ VAS (Table 3). Model 2 explained 20% of the variance in EQ VAS at 15 months post-injury. Bootstrap analysis confirmed all the significant analysis, and all values are presented after bootstrapping in Table 3. Neuroticism and openness to experience were not significantly associated to EQ VAS and were left out of the final model to improve model fit. All the variable inflation factor values were between 1.11 and 1.38, indicating no issues of multicollinearity.

**Table 3:** Model 2: the association between demographics, PPCS, personality traits and EQ VAS (*N* = 86)

Variable	Unstd. Coefficient	Bootstrap Std. err.	*p*-Value	Normal-based C.I. 95%	
Female	-8.16	3.6	**0.024**	-15.23 to -1.09	
Marital status^1^	-2.65	1.27	**0.037**	-5.14 to -0.16	
Children in household^2^	-7.18	3.85	0.062	-14.73 to 0.36	
Baseline RPQ score	-0.24	0.14	0.077	-0.51 to 0.03	
Extraversion	0.36	0.18	**0.049**	0.002 to 0.72	
Agreeableness	0.32	0.18	0.073	-0.03 to 0.66	
Conscientiousness	-0.33	0.26	0.211	-0.84 to 0.19	
Constant	75.72	15.36	0.000	45.6 to 105.83	
Total R^2^		0.26			
Adjusted R^2^		0.20			

^1^Marital status: 0– Married/cohabitating; 1– Single/Living alone, ^2^Children in household: 0– No; 1– Yes. Abbreviations: PPCS, Persistent post-concussion symptoms; EQ VAS, EuroQol Visual Analogue Scale; RPQ, Rivermead Post-Concussion Symptoms Questionnaire. Bold indicates statistical significance

## Discussion

The current study aimed to investigate the association between personality traits and HRQoL 15 months post-injury in patients with mild-to-moderate TBI, using generic and disease-specific measures. The main findings were that more severe PPCS and higher levels of neuroticism and conscientiousness were associated with lower HRQoL, and that extraversion was associated with higher HRQoL irrespective of measure. Female sex and being single/living alone was also significantly associated with lower generic HRQoL.

Our hypothesis was supported by the fact that higher levels of neuroticism were associated with lower HRQoL. To our knowledge, no previous study on TBI has shown this association, but the results are in line with literature outside the TBI field. Huang et al. [33] was the first to systematically review the influence of personality on HRQoL in individuals with various health states (e.g. healthy, aging, and a variety of different illnesses). They identified that neuroticism was more often related to psychosocial aspects of HRQoL and indicated that it was more likely related to psychological rather than physical functioning. Another study showed that general trauma patients at risk for impaired QoL could be identified from a variety of psychological traits at baseline, including neuroticism, but not by sociodemographic or clinical characteristics [53]. Neuroticism has also been associated with higher health care use [54]. In the TBI field, studies have shown that patients with mTBI experience a reduction in HRQoL and lower levels of life satisfaction up to three years after injury [55, 56]. Still, we need more nuanced clinical approaches to identify potential personality-related risk factors in recovery after mTBI.

More severe PPCS were also associated with lower HRQoL, which is in line with the existing literature [2]. Based on the current literature, there is a conceptualization that psychological factors (i.e. neuroticism, anxiety, depression) and PPCS are associated with each other, and lead to worse functional outcome following mTBI [23, 31, 32, 57, 58]. However, exactly how and in which ways these associations unfold remains unclear. It seems reasonable to assume that neuroticism impacts upon mental health and PPCS, since personality traits are considered stable over time in adults [59]. A study which supports this notion [60] found that personality traits associated with negative affectivity (i.e. neuroticism), indirectly predict PPCS through the enhancement of acute somatic complaints. How these psychological factors and PPCS are related to other outcomes (e.g., HRQoL and RTW) are however yet to be detected. Sustaining a mild or moderate TBI can be a risk factor for long-term sickness absence [61, 62] and several studies point to the positive impact of employment on HRQoL and sense of well-being after TBI [12, 63]. We have previously published data showing that there is an indirect association between higher levels of neuroticism and lower RTW via more severe PPCS, in the same sample [42]. Further research is needed to confirm these findings and to additionally investigate if similar mechanisms can be established for HRQoL in patients with TBI. That is, if specific personality traits predict lower HRQoL indirectly via PPCS or other mediating factors.

Higher levels of conscientiousness were associated with lower HRQoL in the current study. Conscientiousness is generally known for its association with positive traits and behaviours, and conscientious individuals typically report higher psychological well-being [64]. This is reflected in the conscientiousness FFM facet structure, which includes competence, orderliness, dutifulness, achievement-motivation, self-discipline, and cautiousness [65]. Despite these generally positive characteristics, conscientiousness has also been related to less favourable outcomes in previous psychological research outside the TBI field. For example a meta-analysis by Fayard and colleagues [66] found that conscientiousness was associated with overall negative affect, and most strongly associated with guilt. Similarly, a study by Carter et al. [67] showed that higher scores on conscientiousness facets was positively related to negative affect in an adult working population. The authors hypothesized that extremely high levels of conscientiousness could have negative implications for psychological well-being, resulting in a curvilinear relationship between conscientiousness and well-being. However, higher levels of conscientiousness are not necessarily related to negative outcomes in isolation, but may depend on contextual factors, such as unemployment [68]. We have also previously found that higher levels of conscientiousness were associated with lower RTW via more severe PPCS, which may support this notion [42].

Higher levels of extraversion– a tendency to experience and exhibit positive affect, warmth, optimism, assertive behaviour, decisive thinking, and sociability [69]– were associated with higher HRQoL. This finding suggests that extroverted individuals may be more resilient to negative life events, which is a new finding in TBI. In line with the finding, the association between extraversion and more adaptive coping styles [70], positive health outcomes [71] and life satisfaction [72] has been described previously in individuals with various health conditions. The literature describes complex relationships between extraversion, social constructs (e.g. social connectedness, - support, -inclusion and -status), and QoL. Connolly and Johansson [73] found that extraversion was strongly associated with life satisfaction (a central component of overall well-being and QoL [74]), but that the relationship between extraversion and life satisfaction was fully mediated by both social inclusion and social status. Further, Lee and colleagues [75] found that the relationship between extraversion and well-being was mediated by social connectedness (i.e. a person’s subjective awareness of being in close relationship with the social world as a whole). Furthermore, Pocnet and colleagues [76] found that perceived social support, as well as extraversion and conscientiousness personality dimensions, were positively linked to life satisfaction and QoL in the general population. Altogether, it seems that both personality characteristics and several social constructs impact upon well-being and QoL. However, more research is needed to better understand these links in the TBI population, where it may help identifying possible protective indicators for individuals’ outcomes (e.g. PPCS, RTW, HRQoL).

Regarding social support, as mentioned above, this can also be relevant to our finding that being single/living alone was associated to lower HRQoL (model 2). This might suggest that people in close relationships (i.e. married or cohabiting) experience more social support, which may serve as an important contributor to life satisfaction. We also found that female sex was related to lower HRQoL measured with EQ VAS. This is in line with a previous study in a large European TBI cohort [77], which also found that women had poorer outcomes on both generic and disease specific measures of HRQoL after mTBI. Evidence indicates that differences in gender roles and identity, alongside biological differences such as hormonal fluctuations, may affect the manner in which different genders experience and report symptoms [77]. Consequently, gender-related factors may impact the results of the outcomes we employ, including HRQoL.

We used a generic and a disease specific measure of HRQoL in this study. Interestingly, the two regression models showed that the independent variables significantly associated with each measure differed, except for extraversion. While generic instruments may capture impairment due to overall clinical presentation [78, 79], disease specific measures are specifically designed to capture the impact of a specific condition on HRQoL. According to the literature, generic instruments are not frequently validated within the TBI field [18] and it is recommended that the EuroQol instruments are used as complementary measures of HRQoL, in addition to health-economic evaluations [80], and not as a substitute for disease-specific instruments [16, 17]. Nevertheless, the mean EQ VAS score in the current sample was considerably lower than the mean scores in the general Norwegian population (53 vs. 77.9) [44].

There were some limitations to this study. The personality traits were measured at 15 months post-injury, which did not allow us test for prediction or mediation. We can therefore not preclude that post-injury functioning may have influenced self-reported personality. While the literature supports that the personality traits of patients with mTBI correspond with those observed in the general population, and that personality characteristics remain stable after mTBI [32, 81], we cannot definitively conclude that experiencing prolonged symptom burden including emotional distress prompted by altered life circumstances, insufficient social support, and uncertainty regarding prognosis, did not exert an influence on the assessment of personality traits. The patients with moderate TBI included in this study were few. Thus, the results should be interpreted with caution in terms of patients with moderate TBI. Furthermore, the findings might not apply to all individuals with mTBI, as the participants were included based on specific criteria (e.g., experiencing persisting symptoms interfering with work participation at 2–3 months post-injury). Yet, the sample is representative of individuals who present to specialized TBI outpatient clinics. The somewhat limited sample size restricted the number of independent variables that could be included in the regression models. The confidence interval in the regression models regarding females was quite wide in our study, indicating greater uncertainty regarding this result. Unfortunately, we were not able to investigate the facets of each of the five personality dimensions because we used the NEO-FFI-3. Having access to facets could have given more knowledge regarding association between more specific personality aspects and outcomes. On the other hand, research has shown that associations between general domains and outcomes can be stronger than facet-level relationships [67]. Lastly, the independent variables accounted for 20 to 30% of the variance in the two models. This suggests that there are additional factors not captured by the current set of independent variables that may significantly contribute to the variance.

Further analysis should investigate the potential non-linearity and interactionality within associations between personality traits and HRQoL. For instance, having particularly low or high levels of personality traits could result in outcomes that deviate from general linear relationships. Moreover, studies in other highly educated populations (medical students/ young doctors) have shown that personality types, for example high levels of neuroticism and conscientiousness, predicted perceived stress [82]. These different combinations of personality traits could also be of interest in TBI recovery and related to QoL, which we plan to look into in future investigations.

## Conclusions

The current study is the first to show associations between personality traits and HRQoL in the first year of recovery after mild-to-moderate TBI. Considering personality factors may be helpful when identifying individual risk and protective factors for outcomes after mild-to-moderate TBI. This is important, not only to improve early effective treatment and reduce individual burden, but also to limit costs to society.

## Electronic supplementary material

Below is the link to the electronic supplementary material.

Supplementary Material 1

## Data Availability

The datasets used and/or analysed during the current study are available from the corresponding author on reasonable request.

## Footnotes

1 The World Health Organization defines quality of life as “the individuals’ perception of their position in life in the context of the culture and value systems in which they live and in relation to their goals, expectations, standards and concerns. It is a broad-ranging concept affected in a complex way by the persons’ physical health, psychological state, level of independence, social relationships, personal beliefs and their relationship to salient features of their environment” [11].

## Declarations

### Abbrevations

CT: Computed Tomography.

EQ VAS: EuroQol Visual Analogue Scale.

FFM: The Five-Factor Model of Personality.

GCS: Glasgow Coma Scale.

HRQoL: Health Related Quality of Life.

LOC: Loss of Consciousness.

mTBI: Mild Traumatic Brain Injury.

NEO-FFI-3: The NEO Five-Factor Inventory-3.

NEO-PI-3: The NEO Personality Inventory-3.

MRI: Magnetic Resonance Imaging.

OUH: Oslo University Hospital.

PPCS: Persistent Post-Concussion Symptoms.

PTA: Posttraumatic Amnesia.

RCT: Randomized Controlled Trial.

RPQ: Rivermead Post Concussion Symptoms Questionnaire.

RTW: Return to Work.

TBI: Traumatic Brain Injury.

QoL: Quality of Life.

QOLIBRI-OS: The Quality of Life after Traumatic Brain Injury Overall scale.
