# Supplementary material for: The relationship between personality traits and health-related quality of life after mild-to-moderate traumatic brain injury
Source: BMC Neurol. 2025 Apr 11;25:157. doi: 10.1186/s12883-025-04153-0 (PMC11987296; doi:10.1186/s12883-025-04153-0)
Supplement: Supplementary file 1 — Supplementary Material 1 [file 12883_2025_4153_MOESM1_ESM.docx]

Descriptive statistics separately for mild and moderate TBI

**Table 1a. Characteristics of the participants with moderate TBI (N = 5).**

Sociodemographic factors Mean/SD N* (%)*

Age, y 45 (10)

Sex, female 2 (40)

Education, y 15 (2,5)

Married/cohabitating 3 (60)

Cause of injury

Falls 3 (60)

Traffic accidents 2 (40)

Intracranial findings on CT/MRI

Yes 4 (80)

Injury severity

LOC Yes 3 (60)

PTA Yes 5 (100)

Extra-cranial injury

Yes 5 (100)

Symptom burden

RPQ total score 20 (16)

QOLIBRI-OS 60 (22)

EQ VAS 56 (17)

Personality traits

Neuroticism 47.6 (8.4)

Extraversion 39.6 (12.6)

Openness to experience 49.6 (12)

Agreeableness 48.2 (8.2)

Conscientiousness 56.8 (3.4)

Abbreviations: CT, computed tomography; LOC, loss of consciousness; MRI, magnetic resonance imaging; PTA, posttraumatic amnesia; RPQ, Rivermead Post-Concussion Symptoms Questionnaire; QOLIBRI-OS, The Quality of Life after Traumatic Brain Injury Overall scale; EQ VAS, EuroQol Visual Analogue Scale.

**Table 1b. Characteristics of the participants with mild TBI (N = 81).**

Sociodemographic factors Mean/SD N* (%)*

Age, y 43 (10)

Sex, female 49 (61)

Education, y 16 (2)

Married/cohabitating 54 (67)

Cause of injury

Falls 34 (43)

Exposure to inanimate objects 17 (21)

Traffic accidents 13 (16)

Sports 12 (15)

Violence 4 (5)

Intracranial findings on CT/MRI

Yes 16 (20)

LOC Yes 28 (35)

PTA Yes 36 (45)

Extra-cranial injury

Yes 36 (44)

Symptom burden

RPQ total score 28 (10)

QOLIBRI-OS 47 (22)

EQ VAS 53 (18)

Personality traits

Neuroticism 49.2 (10.5)

Extraversion 44.7 (10.6)

Openness to experience 47.4 (9)

Agreeableness 54.4 (9.4)

Conscientiousness 55.7 (7.5)

Abbreviations: CT, computed tomography; LOC, loss of consciousness; MRI, magnetic resonance imaging; PTA, posttraumatic amnesia; RPQ, Rivermead Post-Concussion Symptoms Questionnaire; QOLIBRI-OS, The Quality of Life after Traumatic Brain Injury Overall scale; EQ VAS, EuroQol Visual Analogue Scale.

**Table 4**. Correlation between personality traits, QOLIBRI-OS and EQ VAS (N=86)

Neuroticism Extraversion Openness to Agreeableness Conscient- QOLIBRI-OS EQ VAS

experience iousness

Neuroticism 1.0000

Extraversion -0.4276 1.0000

Openness to experience 0.0654 0.0676 1.0000

Agreeableness -0.2563 0.1128 -0.0703 1.0000

Conscientiousness -0.4754 0.3048 -0.0558 0.1631 1.0000

QOLIBRI-OS -0.3311 0.2814 -0.0455 0.1676 -0.0954 1.0000

EQ VAS -0.1546 0.2399 -0.0604 0.1456 -0.0771 0.6713 1.0000

**Q-Q plot for residuals (QOLIBRI-OS)**

**Q-Q plot for residuals (EQ VAS)**
